# Five Numerical Methods to Assess the Ischemic Risks in Dental Pulp and Neuro-Vascular Bundle Under Orthodontic Movements in Intact Periodontium In Vitro

**DOI:** 10.3390/dj13010015

**Published:** 2024-12-27

**Authors:** Radu-Andrei Moga, Cristian Doru Olteanu, Ada Gabriela Delean

**Affiliations:** 1 Department of Cariology, Endodontics and Oral Pathology, School of Dental Medicine, University of Medicine and Pharmacy Iuliu Hatieganu, Str. Motilor 33, 400001 Cluj-Napoca, Romania; ada.delean@umfcluj.ro; 2 Department of Orthodontics, School of Dental Medicine, University of Medicine and Pharmacy Iuliu Hatieganu, Str. Avram Iancu 31, 400083 Cluj-Napoca, Romania

**Keywords:** dental pulp, neuro-vascular bundle, intact periodontium, orthodontic movements, finite elements analysis

## Abstract

**Background/Objectives**: Dental pulp and its neuro-vascular bundle (NVB) are among the least studied dental tissues. This study identified the best method for evaluating ischemic risks in the dental pulp and NVB of healthy lower premolars under orthodontic forces and in intact periodontium. **Methods**: Nine 3D models of the second lower premolar were reconstructed based on the CBCT scans from nine patients. Nine patients (CBCT scan) were subjected to 3 N of intrusion, extrusion, rotation, tipping, and translation. Five numerical methods, Tresca, von Mises (VM), Maximum and Minimum Principal, and hydrostatic pressure were used to biomechanically assess (totaling 225 simulations) the color-coded stress distribution in pulp and NVB. The results (both qualitative and quantitative) were correlated with the physiological maximum hydrostatic pressure (MHP) and known tissular biomechanical behavior. **Results**: All five methods displayed quantitative amounts of stress lower than MHP and did not seem to induce any ischemic risks for the NVB and pulp of healthy intact premolars. Among the five movements, rotation seemed the most stressful, while translation was the least stressful. The NVB displayed higher amounts of stress and tissular deformations than the pulp, seeming to be more exposed to ischemic risks. Higher tissular deformations are visible in NVB during intrusion and extrusion, while pulpal coronal stress is visible only during translation. Only the VM and Tresca methods showed a constant stress display pattern for all five movements. The other three methods displayed various inconsistencies related to the stress distribution pattern. **Conclusions**: Only the Tresca and VM methods can provide correct qualitative and quantitative data for the analysis of dental pulp and NVB. The other three methods are not suitable for the study of the pulp and NVB.

## 1. Introduction

Despite their great importance in the tissular biomechanical behavior, the dental pulp and its neuro-vascular bundle/NVB are among the least studied dental tissues, due to their extremely small dimensions and anatomical complexity [1,2,3,4,5,6,7,8,9]. Their internal anatomical complex micro-architecture rich in circulatory vessels makes them prone to ischemic risks leading to morpho-pathological changes with influence over the physiological functionality and ability to sustain further damage [10,11,12,13,14,15,16,17,18,19,20]. Their individual study is possible only through numerical methods enabling assessment of the stressed areas as well as the tissular deformations during the orthodontic treatment [5,6,7,21,22,23,24,25,26,27,28,29,30,31,32,33,34,35,36,37,38,39,40,41,42]. The in vivo methods do not allow for such an individualized approach, offering only general data of the entire analyzed tissular structure of tooth–periodontal ligament/PDL–bone [43,44,45,46,47,48,49,50].

Although numerical methods were introduced in dental research two decades ago and have been widely used in studies of PDL and implant–bone interactions, their results have often been inconsistent with clinical observations, leading to skepticism and diminished trust in their reliability [43,44,45,46,47,48,49,50]. Nevertheless, in the engineering field, the same method is renowned for its accuracy and is widely used [40]. In an earlier series of stepwise numerical studies [43,44,45,46,47,48,49,50], our team identified and assessed the main accuracy issues and introduced the Tresca method as being more suited for dental studies. By employing this method in both intact and reduced periodontium, under light orthodontic forces and during five orthodontic movements, the results complied with known tissular biomechanical behavior and physiological maximum hydrostatic pressure/MHP of 16–22 KPa [6,7,27,28,32,41,42,46,47,51]. The reported accuracy issues were related to the incorrect use of the analyzed material-based method, anatomically inaccurate 3D models, incorrect boundary assumptions, and lack of correlation with MHP values [40,43,44,45,46,47,48,49,50].

One of the factors triggering orthodontic movement is represented by the circulatory disturbances induced by the orthodontic force [1,2,46]. If the MHP pressure is exceeded for long periods of time, ischemia is induced, leading to resorptive–degenerative processes and tissular necrosis. In intact periodontium, the healthy intact dental tissues possess a good absorption–dissipation ability [43,44,45,46,47,48,49,50], displaying little damage for a higher amount of orthodontic force applied for limited periods of time [2,5,6,7,32,45,46,52]. This ability is modified and diminished in the situation of a previous tissular trauma/injury (i.e., occlusal trauma for NVB [5,6,7,8,9] and direct-indirect coronal pulp capping for dental pulp [10,11,12,13,14,15,16,17,18,19,20]) [5,6,7,8,9,20,31,32,53,54,55,56,57,58]. These are not clinically visible, while their ischemic consequences appear during the orthodontic movements when the irreversible mechanism has already started [5,6,7,8,10,11,12,13,14,15,16,17,18,19,20,31,32,54,55,56,57,58]. Nevertheless, by displaying the stressed areas the numerical analysis can anticipate some of these ischemic risks and avoid them by carefully selecting the amount of applied load.

It is accepted that up to 1 N light orthodontic forces are usually safe for intact periodontium [43,44,45,46,59]. However, the optimal amount remains a subject of debate. The numerical methods used in dental studies [21,22,23,24,25,26,27,28,30,33,34,35,36,37,38,39,40,41,42,52,60] are maximum tensile and minimum-compressive principal, von Mises/VM-overall, Tresca-shear, which was only recently introduced, and hydrostatic pressure-liquids/gas. The main issue regarding dental studies is related to the fact that despite using a similar methodology as in engineering fields, the results suffer from major accuracy issues [40,43,44,45,46,47,48,49,50]. There are many contradicting and confusing reports regarding the optimal amount of force or/and the most stressful movement without displaying a coherent image of the tissular biomechanical behavior such as the clinical reality. Thus, reports of optimal forces up to 3 N, with rotation or intrusion being the most stressful, with a high amount of stress in the apical third of PDL holding NVB exceeding the MHP even for light forces, associated with insignificant PDL cervical third stress for orthodontic movements, are some of these inconsistencies [27,28,41,42,52,60] that contradicted the clinical reality [1,2,3,4]. Additionally, there are some reports related to the poor quality of in vivo studies [1,3,31].

To settle the results accuracy problem of employing different numerical methods for the analysis of pulp and NVB, a comparative study of multiple criteria is needed. To assess if a higher force of 3 N is prone to induce ischemic risks in healthy intact tissues and periodontium during orthodontic movements is also desirable for both clinician and researcher.

The aims of this study were to identify the most effective method for assessing the risk of ischemia during orthodontic treatment by comparison of five methods for analyzing the biomechanical behavior of the dental pulp and its neuro-vascular bundle (NVB) in healthy lower premolars with intact periodontium, subjected to 3 N force orthodontic movements.

## 2. Materials and Methods

Our study is part of a larger stepwise research with clinical protocol 158/02.04.2018 assessing the orthodontic forces effects over dental tissues in various degrees of periodontal loss [43,44,45,46,47,48,49,50].

Nine intact periodontium 3D models of healthy second lower premolars obtained from nine patients (4 males/5 females, mean age 29.81 ± 1.45), were used in 225 numerical simulations.

The inclusion criteria were: no missing teeth or malposition in the investigated area; intact lower premolar free of endodontic, filling, and crown treatments; bone loss limited to 1–2 mm; no inflamed periodontium and proper oral hygiene; and orthodontic treatment indication. The exclusion criteria were: more than 2 mm bone loss; particular root geometry involving non-fused double root, angulated root, and extreme curvature; abnormal crown shape with multiple cusps; root surface defects with external root resorption; radiologically visible bone defects; abnormal pulp chamber and root canals with internal resorption; and poor oral hygiene after inclusion with visible signs of inflammation.

**Figure 1:**
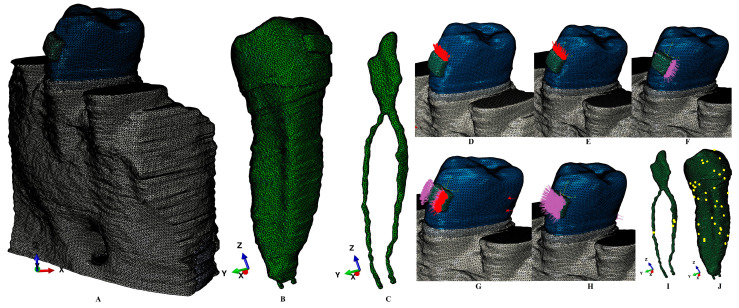
Example of 3D mesh model: (**A**)—2nd lower right premolar model with intact periodontium, (**B**)—second lower premolar with base of the bracket and NVB, (**C**)—dental pulp and NVB, applied vectors: (**D**)—extrusion, (**E**)—intrusion, (**F**)—translation, (**G**)—rotation, (**H**)—tipping, (**I**)—dental pulp and NVB mesh with elements warnings, (**J**)—second lower premolar with mesh elements warnings.

The premolar mandibular region was radiologically investigated by using a CBCT (ProMax 3DS, Planmeca, Helsinki, Finland; voxel size of 0.075 mm). The tissular selection and reconstruction process was performed using AMIRA 5.4.0 (Visage Imaging Inc., Andover, Andover, MA, USA). The manual reconstruction process was preferred due to the tissular complexity and anatomical small dimensions. Each tissular component was individually identified and reconstructed: enamel, dentine, dental pulp, neuro-vascular bundle/NVB, trabecular and cortical bone, periodontal ligament/PDL (Figure 1). The cementum could not be clearly separated from the dentine and was reconstructed as dentine due to similar physical properties (Table 1). PDL has a variable thickness of 0.15–0.225 mm containing premolar’s NVB. Each of the nine models had limited bone loss of up to 2 mm. Only the second lower premolar was guarded. The rest of the root sockets were filled with trabecular and cortical bone. A tissular reconstruction of missing bone and PDL was performed obtaining thus nine 3D models with intact periodontium. A stainless steel bracket base was reconstructed on the vestibular coronal side of the enamel crown.

The mesh had 5.06–6.05 million C3D4 tetrahedral elements, 0.97–1.07 million nodes, and a global element size of 0.08–0.116 mm (Figure 1). The mesh had no element errors, and only a limited number of element warnings (e.g., Figure 1F, tooth mesh, 39 element warnings, 0.00589% for a total of 661,137 elements; Figure 1G, pulp-NVB mesh, 4 element warnings, 0.0158% for a total of 25,252 elements). Element warnings signaled extremely small discontinuities in non-essential regions with no influence over biomechanical behavior.

The numerical analysis was performed using ABAQUS 6.13-1 (Dassault Systèmes Simulia Corp., Maastricht, The Netherlands), and employed five methods/failure criteria used by current research flow dental studies: von Mises (maximum overall), Tresca (maximum shear), maximum principal (maximum tensile), minimum principal (maximum compressive) and hydrostatic pressure (liquids/gas). The boundary assumptions were selected as zero displacements based on the models, isotropy, linear-elasticity, and homogeneity/non-homogeneity, as in the current research flow. By using each of these five methods, five orthodontic movements (intrusion, extrusion, rotation, tipping, and translation) under 3 N were assessed. To have a precise amount of force, the appliance surface was carefully taken into consideration. The results were displayed as color-coded various intensity (red-orange high, yellow-green moderate, and blue low) stresses, in the dental pulp and NVB, displaying also the tissular deformations. The reported stress amounts were correlated with physiological 16–22 KPa of MHP [27,28,41,42,43,44,45,46,47,48,49,50] to be able to assess the ischemic risks [43,44,45,46,47,48,49,50]. The results were also correlated with known clinical tissular biomechanical behavior.

## 3. Results

**Figure 2:**
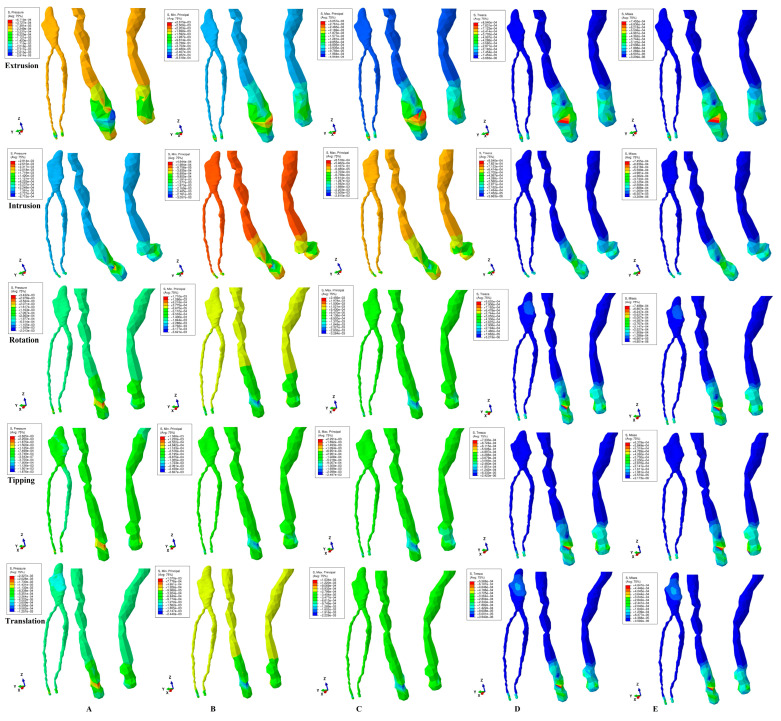
Comparative stress distribution for 3 N in intact periodontium and for the five movements: (**A**)—Hydrostatic Pressure, (**B**)—Minimum Principal, (**C**)—Maximum Principal, (**D**)—Tresca, (**E**)—Von Mises.

Based on tissular biomechanical behavior, (Figure 2), the five methods and orthodontic movements showed that the highest amount of tissular stress was present in the neuro-apical vascular bundle (Table 2). Both coronal and radicular pulp showed extremely low amounts of stress. All quantitative results were lower than the physiological maximum hydrostatic pressure of 16 KPa, showing that there were no ischemic risks under 3 N for intact periodontium and healthy premolars. The NVB stress was 5.6–9.5 times higher than pulpal stress. Thus, NVB structure seems to be more exposed to ischemic risks during orthodontic movements than dental pulp.

Quantitatively, the five methods displayed rotation as the most stressful movement for both NVB and dental pulp and prone to higher ischemic risks, closely followed by tipping, intrusion, and extrusion (Table 2).

The Tresca maximum shear (Figure 2D) and VM maximum overall (Figure 2E) methods displayed similar qualitative color-coded stress display for the five orthodontic movements, probably due to the special design for ductile resemblance tissues. The minimum intensity blue color-coded stress was shown during the five movements in the entire dental pulp. The NVB stress was displayed as medium in most extended areas and higher intensity in limited area stresses, as expected according to the clinical knowledge. In terms of tissular biomechanical behavior, the intrusion and extrusion showed higher deformations of the NVB region, while translation showed coronal pulp (i.e., vestibular, mesial, and distal sides) stress areas. These aspects signal that these above-mentioned movements are biomechanically more challenging for the concerning tissues, especially if these tissues are previously injured/traumatized. Quantitatively, the amounts of stress were the lowest among the five methods, 18.6–228.5 times lower than MHP (Table 2). The quantitative differences between the two methods of 1.11–1.15 are within the specified range, with VM being a little bit lower than Tresca [43,44,45,46,47,48,49,50].

Qualitatively, maximum principal (maximum tensile, Figure 2C), minimum principal (maximum compressive, Figure 2B), and pressure (hydrostatic pressure, Figure 2A) showed various inconsistencies regarding the color-coded stress display in both NVB and pulp, with almost similar colors in both structures. This display pattern suggested similar risks for dental pulp as for the NVB even though biomechanically the pulp is protected by the dental pulp chamber and root canals made of dentine, while NVB is held in the apical third of periodontal ligament suffering more stress and deformation with biomechanical adsorption-dissipation role. The displayed biomechanical behavior seems to contradict both clinical knowledge as well as the quantitative amounts of stress display (Table 2), which clearly shows that NVB stress is much higher than pulpal one. These contradictions are due to the design specifications of each of the three methods, brittle as for maximum and minimum principal, and liquids/gas as for hydrostatic pressure. Nevertheless, the tissular NVB deformations are visible for the intrusion and extrusion in all three methods. The pressure method was the only one to show, during translation, the same coronal pulpal stress as VM and Tresca.

Quantitatively, the pressure method displays negative coronal pulp values for extrusion and translation and positive ones for the other three movements, without scientific explanation. No negative compressive stresses should clinically be present in this protected area. In a similar manner, the maximum and minimum principal stresses have negative compressive signs for the coronal pulp for extrusion, tipping, and translation, with the same above-mentioned observations. Another bizarre issue is represented by the higher pulpal coronal stress of 1.13 KPa for tipping and 1.61 KPa for rotation, in the case of the hydrostatic pressure. Thus, it discontinued the pattern provided by the other methods (i.e., around 0.22–0.34 KPa, seven times lower than that previously mentioned), without any biomechanical explanation. The highest quantitative amount of stress among the five movements was displayed by the minimum principal (4.38–80 times lower than MHP) and hydrostatic pressure (4.66–72.7 times lower than MHP). Based on the above, it seems that these three methods are not as accurate and reliable as the VM and Tresca methods when describing the NVB and dental pulpal behavior.

## 4. Discussion

This study aimed to identify the most effective method for assessing the risk of ischemia during orthodontic treatment by the comparison of five methods for analyzing the biomechanical behavior of the dental pulp and its neuro-vascular bundle (NVB) in healthy lower premolars with intact periodontium, subjected to 3 N force orthodontic movements.

No similar studies were found in the current research flow, despite the importance of the subject for the orthodontic treatment plan.

This study involved 225 numerical simulations in healthy intact lower premolars and periodontium.

Four of the employed methods, von Mises, maximum and minimum principal, and hydrostatic pressure were currently used in the dental numerical studies [21,22,23,24,25,26,27,28,30,33,34,35,36,37,38,39,40,41,42,52,60]. The Tresca method was only recently introduced by our team’s research [43,44,45,46,47,49,50]. It must be emphasized that both NVB and dental pulp can be individually studied only by using numerical studies, which are difficult to perform, explaining the reduced number of available reports [1,2,3,4,5,6,7,8,9]. General information regarding dental tissues can be obtained through in vivo studies but lacks the individual focus on dental pulp and NVB [1,2,3,4,5,6,7,8,9].

Our results showed that only the Tresca and von Mises methods can provide accurate qualitative and quantitative results, in agreement with known clinical biomechanical behavior. These are related to the fact that both methods were originally designed for ductile-like resemblance materials. The dental tissues were seen as ductile resemblance materials but with a certain brittle flow mode [43,44,45,46,47,48,49,50]. The other three criteria were designed for brittle materials (maximum and minimum principal) and liquids/gas (hydrostatic pressure). There are no previous studies in the current research flow to perform such correlations between different methods except our previous [43,44,45,46,47,48,49,50].

In a previous report [46], a similar correlation was performed for the light orthodontic forces of 0.5 N, similarly reporting Tresca and von Mises as being the most suited for pulp and NVB. Moreover, the Tresca method seems quantitatively more accurate due to some aspects related to the analyzed material suitability. Even though both VM and Tresca are designed for ductile-like materials that, when subjected to loads, deform without breaking, and recover the original form when the force ceases, VM is specific for homogenous while Tresca is for non-homogenous materials [43,44,45,46,47,48,49,50]. The living tissues are non-homogenous materials, thus, quantitatively Tresca seems to be closer to clinical reality [43,44,45,46,47,48,49,50]. Nevertheless, quantitatively, the difference is only around 1.15 times, meeting the engineering field interval, and due to extremely small, displayed stresses, both methods can be seen as suited. Moreover, the qualitative stress display is similar for both methods (Figure 2). These are in line with our previous reports [43,44,45,46,47,48,49,50].

The other three methods, despite the multitude of studies [21,22,23,24,25,26,27,28,30,41,42,52,60] are fundamentally incorrect due to the suitability issues [43,44,45,46,47,48,49,50]. The maximum and minimum principal methods are suited for brittle-like and homogenous materials. Physically and mechanically, a brittle material, when subjected to a load, suffers from an extremely small deformation and directly breaks, with no recovery of the original form [40]. The living tissues do not follow this mechanical behavior [40]. However, from the mechanical point of view, the methodological reasoning behind their use is understandable since in orthodontic treatment the deformations and displacements are extremely small and limited, as is the load amount, never arriving at the breaking point [43,44,45,46,47,48,49,50]. However, their working protocol and algorithm differs from that of the ductile-like materials while their described tissular stress display is fundamentally different [40]. Moreover, their quantitative values are four times higher than those provided by Tresca and VM. These observations agree with our previous reports [43,44,45,46,47,48,49,50].

The hydrostatic pressure method was designed for liquids/gas with no shear stress algorithm since the liquids do not display this type of stress in their mechanics. The similarity with living tissues due to the high percentage of water contained by these tissues is fundamentally incorrect since their internal anatomical micro-architecture is different from that of a liquid, as is their biomechanical behavior. Moreover, when comparing the stress display with that of a solid material there are significant visible differences while the quantitative results are extremely high (e.g., almost four times higher than T/VM methods). These are confirmed by our previous research [43,44,45,46,47,48,49,50].

The engineering field used forces are much higher than 3 N. In dental tissues, 3 N induces extremely small deformations and displacements. The five methods similarly reported rotation to be the most stressful movement, with NVB being more prone to ischemic risks than pulp but with no real clinical anticipated risks for healthy intact tissues, in agreement with our previous [43,44,45,46,47,48,49,50], other reports and clinically known tissular biomechanical behavior [1,3,5,6,7,8,20,31,32,40,54,55,56,57,58,59]. The five methods displayed visible NVB deformation for intrusion and extrusion showing tissular susceptibility to ischemic risks, which is of importance if previous occlusal trauma is present [5,6,7,10,11,12,13,14,15,16,17,18,19,20], in agreement with known clinical data [10,11,12,13,14,15,16,17,18,19,20]. Moreover, there is pulpal coronal stress visible during translation through Tresca and VM, and barely through hydrostatic pressure, with no real impact or ischemic risk susceptibility for healthy intact tissues. However, it could be of importance if the pulpal tissue was previously traumatized/injured during earlier dental treatment through direct/indirect pulp capping [5,6,7,8,20,31,32,54,55,56,57,58] in agreement with clinical data [9,53]. Thus, since all quantitative results are lower than MHP, 3 N of applied force seems free of any ischemic risks in healthy intact tissues and periodontium, in line with our previous reports [43,44,45,46,47,48,49,50].

All five methods displayed rotation as the most stressful movement for the pulp and NVB, in agreement with our previous studies [43,44,45,46,47,48,49,50], other studies [10,11,12,13,14,15,16,17,18,19,20], and Wu et al.’s reports [41,42,60]. Intrusion and extrusion closely followed the rotation as stressful movements, in agreement with Minch et al. [52] and Hofman et al. [27,28] regarding the intrusion as stressful movement in intact periodontium.

There is only limited data available in the current research flow regarding the dental pulp and NVB due to difficulties in radiologically identifying, selecting, and reconstructing these complex and small tissues [43,44,45,46,47,48,49,50]. Nevertheless, for validation reasons and since biomechanically NVB is held in the apical third of PDL with absorption–dissipation function [43,44,45,46,47,48,49,50], correlations with PDL numerical studies are possible, as well as with the physiological maximum hydrostatic pressure of 16 KPa present at this level. However, the available numerical method studies [21,22,23,24,25,26,27,28,29,30,41,42,52,60] did not hold for the herein approach regarding the engineering field requirements mandatory for accurate results. Thus, provided reports that sometimes contradicted clinical knowledge, creating mistrust regarding the method’s accuracy despite its engineering field-renowned accuracy. There are multiple intact periodontium PDL numerical studies available, employing few orthodontic movements, one/two [27,28,30] and anatomically simplified 3D models (i.e., upper 1st premolar [27,28,30,41,42]; canine [41,42,60]; 1st molar and incisor [22,23,24,25,26,41]). Most of these studies assessed intact periodontium [27,28,30,41,42,60] and only a few investigated variable levels of reduced periodontium [24,25]. These studies employed hydrostatic pressure [27,28,30,41,42,60], maximum and/or minimum principal [21,22,23,24,25], and VM [21,26,30] without acknowledging the studied material type and MHP [21,22,23,24,25,26]. The boundary assumptions also included non-linearity [21,22,23] and linearity [24,25,26]. Some of the reported results were higher than MHP [21,22,23,24,25]. They used forces of 1–6 N [27,28,41,42,60], and hydrostatic stress [27,28,30] as single criteria for the study of PDL, contradicting the clinical knowledge. As a major issue related to modeling, PDL studies [21,22,23,24,25,26,27,28,30,41,42,52,60] did not reconstruct the NVB component. Thus, despite the existence of quantitative data, sometimes their scientific value is limited due to the above-mentioned methodological issues, reports of PDL apical third amounts of stress exceeding MHP for light orthodontic forces [27,28,41,42,60] and are clinically incorrect. Other reports provided qualitative stress display issues, reporting extremely high PDL apical third stress and insignificant cervical third stress [41,42,60], biomechanically fundamentally incorrect [5,6,7,22,23,31,32,59]. It must be emphasized that all the above-mentioned accuracy problems are related to the selection of the improper numerical method designed for brittle-like materials or liquids for studying a ductile resembling material as dental tissues [40]. Our herein as well as previous [43,44,45,46,47,49,50,51] reports proved the qualitative and quantitative differences when comparing those different methods.

All numerical studies, despite the many advantages, have some limits related to the fact that they cannot completely reproduce the tissular biomechanical behavior and the anatomical internal micro-architecture, while their results are highly influenced by study method type and boundary assumptions correlated with physical properties and anatomical correctness. One is related to the fact that it cannot completely simulate the clinical situations, since clinically rarely there are pure movements and mostly associations. Based on this, the clinical amounts of stress found in NVB and pulp could be slightly lower but with no impact on the results’ accuracy. Another accuracy limit is represented by the correct use of the method as requested by the engineering field methodology: anatomical correct 3D models, material-based type method, and boundary assumptions [43,44,45,46,47,48,49,50], as this study proved. Anatomical accuracy is directly influenced by the number of elements and nodes, 6.05 million tetrahedral elements, 1.07 million nodes, global element size of 0.08–0.116 mm, 40–12,731 times more elements, 4.4–1463 times more nodes than previous studies [5,6,7,21,22,23,24,25,26,27,28,29,30,31,32]. It is also influenced by the type of the 3D models, CBCT-based vs. idealized simplified models, by the number of models with higher sample size vs. only one and the total number of simulations of 225 herein vs. few [21,22,23,24,25,26,27,28,30,33,34,35,36,37,38,39,40,41,42,52,60]. The sample size for a numerical study method is usually one specific to the engineering field, thus, most of the dental studies used one patient/one model and few simulations [22,23,26,33,34,35,36,37,38,39,40]. It must be emphasized that this single sample size is due to multiple changing variable possibilities providing different situations and results. Thus, starting from these above-mentioned, new numerical studies of dental tissues must be performed, since most of the current ones do not meet these accuracy requirements [1,3,31,40].

## 5. Conclusions

1. The five methods displayed lower MHP amounts of stress for 3 N, seeming not to induce any ischemic risks for NVB and pulp of healthy intact premolars and periodontium.
2. Only the Tresca and VM methods can provide correct qualitative and quantitative data for the analysis of dental pulp and NVB. The other three methods are not suitable for pulp and NVB study.
3. Among the five movements, rotation seems the most stressful, while translation is the least stressful.
4. The NVB displayed higher amounts of stress and tissular deformations than the pulp, seeming to be more exposed to ischemic risks.
5. Higher tissular deformations are visible in NVB during intrusion and extrusion, while pulpal coronal stress is visible only during translation.

## 6. Practical Implications

Our study is the first to approach the difference issue when using different methods of tissular biomechanical behavioral study. No similar studies were found except for our previous research. Moreover, only a few reports studied dental pulp and none the NVB. By using five methods, the comparative study of dental pulp and NVB biomechanical behavior under orthodontic forces, movements, and in intact periodontium provided not only a clear methodology for obtaining accurate results but also a complete set of data necessary for the clinical planning phase of the orthodontic treatment (i.e., amount of force, movement, tissular and periodontal status, appliance time). To know that 3 N of force has no ischemic risks over dental pulp and NVB in any of the five orthodontic movements is of extreme importance for the clinical practitioner. Moreover, by proving the differences when using different methods for the study of pulp and NVB, it also provides both the clinician and the researcher with new data needed to improve the numerical studies methodology.

## Figures and Tables

**Table 1:** Physical properties of materials.

Materials	Young’s Modulus, E (GPa)	Poisson Ratio, ʋ	Refs.
Enamel	80	0.33	[43,44,45,46,47,48,49,50]
Dentin/Cementum	18.6	0.31	[43,44,45,46,47,48,49,50]
Pulp and NVB	0.0021	0.45	[43,44,45,46,47,48,49,50]
PDL	0.0667	0.49	[43,44,45,46,47,48,49,50]
Cortical bone	14.5	0.323	[43,44,45,46,47,48,49,50]
Trabecular bone	1.37	0.3	[43,44,45,46,47,48,49,50]
Stainless steel bracket (Cr-Co)	218	0.33	[43,44,45,46,47,48,49,50]

**Table 2:** Maximum stress average values (KPa) produced by 3 in NVB and coronal pulp.

Resorption (mm)			Extrusion	Intrusion	Rotation	Tipping	Translation
	**Tresca**	NVB	**0.72**	**0.70**	**0.86**	**0.73**	**0.56**
		c	**0.07**	**0.07**	**0.15**	**0.12**	**0.10**
	**VM**	NVB	**0.56**	**0.56**	**0.75**	**0.64**	**0.48**
		c	**0.07**	**0.07**	**0.13**	**0.11**	**0.12**
	Pressure	NVB	−2.62	2.62	3.43	2.63	2.32
		c	−0.22	0.22	1.61	1.13	−0.34
	S1	NVB	2.53	−2.22	−3.33	−2.56	−1.97
		c	−0.50	0.25	0.56	−0.50	0.25
	S3	NVB	2.81	−2.76	−3.62	−2.83	−2.44
		c	−0.24	0.20	0.38	−0.25	−0.10

NVB—neuro-vascular bundle, c—coronal pulp.

## Data Availability

The original contributions presented in the study are included in the article, further inquiries can be directed to the corresponding author.
